# Digital Treatment Paths for Substance Use Disorders (SUDs)

**DOI:** 10.3390/ijerph19127322

**Published:** 2022-06-15

**Authors:** Natale Salvatore Bonfiglio, Maria Lidia Mascia, Maria Pietronilla Penna

**Affiliations:** 1 Department of Psychology “Renzo Canestrari”, University of Bologna, 40126 Bologna, Italy; s.bonfiglio@unibo.it; 2 Department of Pedagogy, Psychology, Philosophy, University of Cagliari, 09123 Cagliari, Italy; penna@unica.it

Today, there is a considerable expansion in the number of new digital tools and systems for mental health assessment, intervention, support, prevention, and treatment. Many studies highlight the effectiveness and importance of digital tools in various areas of healthcare and preventive interventions for issues such as substance use disorders, mental health disorders, and disorders with comorbidities.

In 2018, an estimated 269 million people worldwide have used illicit drugs at least once in the past year, corresponding to 5.4% of the world population aged 15 to 64. Based on these data, the global number of people who will use illicit drugs will increase by 11% by 2030, reaching 299 million people. This projection reflects population growth only [1].

Many studies suggest that adolescence and young adulthood (15–34 years old) are critical periods for drug use initiation [2]. Therefore, treatment and preventive interventions directly involving these age groups would help in inhibit the estimated growth of substance use.

In general, due to various obstacles, only about 20% of people suffering from substance use disorders can access available treatments [3]. Additionally, almost one in five adults suffer from a mental disorder at some point in their lives [4] and nearly 13% of drug users are estimated to suffer from mental disorders, meaning that their drug use is harmful to the point of drug addiction and/or need for treatment [2]. Studies have also shown that problems related to substance use tend to persist for patients with severe mental illnesses [5]. People who suffer from mental problems and use drugs are unlikely to access treatment services because of stigma, lack of treatment services in their area, and so on. Therefore, it is important to reach as many people as possible to give them pathways to recovery. Digital services and tools could facilitate access to healthcare, especially for adolescents and young adults who can be reached because they are familiar with new technologies or are more technologically literate and mainly involved in using new technologies [6].

One solution to fill possible gaps and create easier access to treatment, especially for younger people with substance abuse problems, can be found in the use of technology in healthcare. The convergence of digital technologies with the fields of healthcare, referred to as digital health, has enabled the development and deployment of technological tools to monitor and promote accessible healthcare. The field of digital health is active, innovative, and constantly evolving, offering a rich array of digital tools aimed at providing therapeutic treatments and ensuring effective screening, monitoring, diagnosis, and treatment. Digital health employs smartphone applications, chat bots, and wearable devices such as bracelets, patches, glasses equipped with artificial intelligence, and other digital tools for collecting physiological parameters.

Different types of interventions using digital tools are growing in popularity. Digital technology has rapidly pervaded and transformed the daily lives of people as the capabilities and reach of technology have expanded. There is an accompanying proliferation of digital technologies specifically developed for use in patient care [7]. Data support the use of digital interventions for substance abuse disorders [8,9], particularly because of how internet-based technologies have the capacity to merge the efficacy of evidence-based treatments with the advantages of wide-reaching interventions to improve access to substance use care [10]. Some treatments are fully automated and independent of human support (self-directed or unguided interventions), while others require the involvement of a therapist or technician (guided interventions) [11]. It is possible to use software and digital platforms structured as training programs to carry out more focused interventions for specific substance use issues and symptoms, such as cravings [12].

The advantages of these tools have been emphasised during the COVID-19 pandemic, during which the difficulty of accessing mental health services has increased. Digital solutions have received more interest and have offered accessible, affordable, personalised, and other attractive benefits of digital health interventions in many areas [13].

The most common mental health intervention strategies make use of smartphone apps [14]. They are growing steadily, as smartphones are becoming increasingly prevalent in developing countries and are already used by about three billion people around the world. Through apps, it is possible to cover all phases of psychological care, including prevention, diagnosis, treatment, face-to-face therapy, and relapse prevention [15].

Sophisticated technological tools, such as virtual reality (VR) and augmented reality (AR), are also being successfully used for the assessment and treatment of mental disorders [16]. They have the advantage of immersing the user in highly realistic environments to reproduce treatment-specific conditions and realities.

Treatments delivered via digital platforms have the advantage of being largely accessible at different groups of people within a population [17]. Digital health interventions present many advantages, such as interactivity, 24/7 availability that allows “on-demand” access to therapeutic support, self-directed software tools that can overcome some of the striking disparities in treatment access, and consistently high-quality treatment that transcends borders [18]. Digital interventions can teach people effective and scientifically validated skills to recognise and change unhealthy thoughts and behaviours and provide tools to help people apply these skills to their everyday lives.

The application of digital technologies to better assess, understand, and treat substance use disorders is a particularly active area of scientific research [17]. In general, the potential advantages of technology in enhancing healthcare and mental health care to treat substance use disorders and their psychiatric comorbidities have been recognised and appreciated in several recent reviews [19,20,21,22,23].

Moreover, a body of literature and some specific meta-analyses underline the effectiveness of these approaches to health and mental health. Fu and colleagues [24] show the effectiveness of digital psychological interventions (delivered in several formats, including via websites, smartphone apps, computer, audio-devices, and text messages) for mental health problems (depression and substance misuse comorbidity). The authors obtained a pooled Hedge’s g effect size of 0.60, comparing the effects of digital psychological interventions with a control group, with a substantial heterogeneity between studies.

Effectiveness and treatment moderators of internet interventions for adult problem drinking are demonstrated in the meta-analysis conducted by Riper and colleagues [25]. The meta-analysis revealed a small significant difference in mean weekly substance use in favor of internet-assisted intervention participants as compared controls, with a pooled Hedge’s g effect size of 0.26 and a moderate heterogeneity, indicating that the effect was greater in some trials than in others.

Oh and colleagues [26] highlight the potential for digital interventions to prevent alcohol consumption among pregnant women and women planning to become pregnant, thanks to text messaging service or automated internet intervention or interactive sessions. The authors reported a decrease in alcohol consumption during pregnancy compared to the control group with an Odds Ratio (OR) of 0.62. Moreover, computer-based interventions (OR of 0.59) and text messaging (OR of 0.8) proved to be effective for preventing alcohol consumption using a stratified analysis aimed at examining the influence of different intervention platforms.

Boumparis and colleagues [3] published a meta-analysis about the effectiveness of short- and long-term effects of digital prevention and treatment interventions for cannabis use reduction. The authors reported that prevention interventions compared to non-active control conditions showed a small significant effect (pooled Hedge’s g = 0.33) on cannabis use at post-treatment, with high heterogeneity.

Other meta-analyses confirm the effectiveness of stand-alone digital technology interventions for screening and treating common mental disorders [27] and of stand-alone smartphone apps for mental health, showing also significant effects on smoking of apps compared to control groups [28]. Sin and colleagues [27] reported how, comparing digital interventions with inactive controls or other comparable interventions, digital interventions showed Standard Mean Difference (SMD) of −0.30 for depression, −0.37 for anxiety, −0.43 for stress, −0.16 for social functioning, and 0.40 for wellbeing. Weisel and colleagues (2019) reported a significant effect of smartphone apps compared to controls for depression with a pooled Hedge’s g = 0.33 and moderate heterogeneity.

Thanks to the widespread use of technologies, the development, design, and use of health and digital therapeutic health tools have become possible. These tools can prove useful in overcoming barriers related to access to healthcare, especially among younger people. They represent a solution for remote monitoring and real-time health surveillance. In addition, digital health tools can act as a bridge between users and health systems, which promotes greater awareness of patients’ health statuses and provides reliable and more easily accessible information. Compared with traditional clinical practices, digital health tools and resources result in lower costs. Finally, by allowing people to access healthcare from wherever they choose, these tools can help those in need overcome shame and social stigma and break down the barriers to asking for help. Due to the considerable heterogeneity observed in the literature about digitals mental health tools, more studies are needed to demonstrate the effectiveness of digital health and to implement standardised digital intervention programs to improve their reproducibility and efficiency [24].
